# The achievements and challenges of occupational health research: Looking back and ahead

**DOI:** 10.5271/sjweh.4136

**Published:** 2024-01-01

**Authors:** Reiner Rugulies, Alex Burdorf

**Affiliations:** National Research Centre for the Working Environment (NFA)and Department of Public Health, University of Copenhagen,Copenhagen, Denmark. [email: rer@nfa.dk]; Department of Public Health, Erasmus Medical Center Rotterdam, Rotterdam, The Netherlands. [email: a.burdorf@erasmusmc.nl]

With this first issue of 2024, we kick off the celebration of 50 years of publishing research in the *Scandinavian Journal of Work, Environment & Health*. In January 1975, the inaugural issue of the Journal was published (read it here: www.sjweh.fi/issue/274). We are delighted that 50 years later not only are we still around, but we have retained our special position in the science publication business as an independent journal that is not owned by a commercial publishing house. We are grateful that our not-for-profit publisher, the Nordic Association of Occupational Safety and Health (NOROSH), has ensured this independence. And we are proud to belong to a community of authors, reviewers, editors, international advisory board members, and, of course, readers that is committed to research excellence and has carried this journal for now half a century. Thank you!

We want to celebrate our anniversary by looking both back and ahead. In each issue of 2024, we will include an invited 50-year-anniversary article, authored by leading researchers in the field. In this first issue, we take a look at ourselves, reflecting on work environment exposures and health outcomes that have emerged as well as those that have vanished from the pages in the Journal. And we take a look at those papers that have generated the greatest interest among our readers (1).

In the coming year, various authors will take a look at the research field, that is, they will discuss specific work environment conditions and health outcomes. This will include exposures as diverse as asbestos and the psychosocial work environment and outcomes such as musculoskeletal disorders and occupational cancers. We will in particular examine the successes and failures with regard to these exposures and outcomes. Has occupational health research made a difference? Are there success stories where our research has helped to protect and improve workers’ health and thereby contributed to a better population health? Did we provide solid evidence on health hazardous, health-protecting, and health enhancing working conditions and did this result in changes at the workplace? Or did we fail? Was our research not good enough to provide sufficient evidence for action? Or was our research good enough but action still did not happen? And what can we do in the future to improve? How can we do research better and thus make a difference in society?

We are looking forward to the answers to these questions in the anniversary-related papers, which are incidently not yet written, so we do not know what they will tell us. Our guess is, though, that there are no easy answers and a lot of work still remains ahead of us. We and others have recently argued in a Discussion Paper series on “Work and Health” in *The Lancet* that “major gains in population health and reductions in health disparities can be made by an increased focus on improving the work environment.” (2). However, as we also argue in the series, there is still a long way to go in realizing the potential that good work has for better population health (2–4).

Our objective with 50-year SJWEH anniversary series is that, by the end of the year, the occupational health community will have become more knowledgeable about what went well and not so well in our research field and that we feel confident where to go next. To engage the community further in this discussion, we are planning a half-day in-person and online symposium on 4 October 2024. We are still working on the exact format and content of the symposium. Please stay tuned on LinkedIn and follow the updates in our newsletter.
